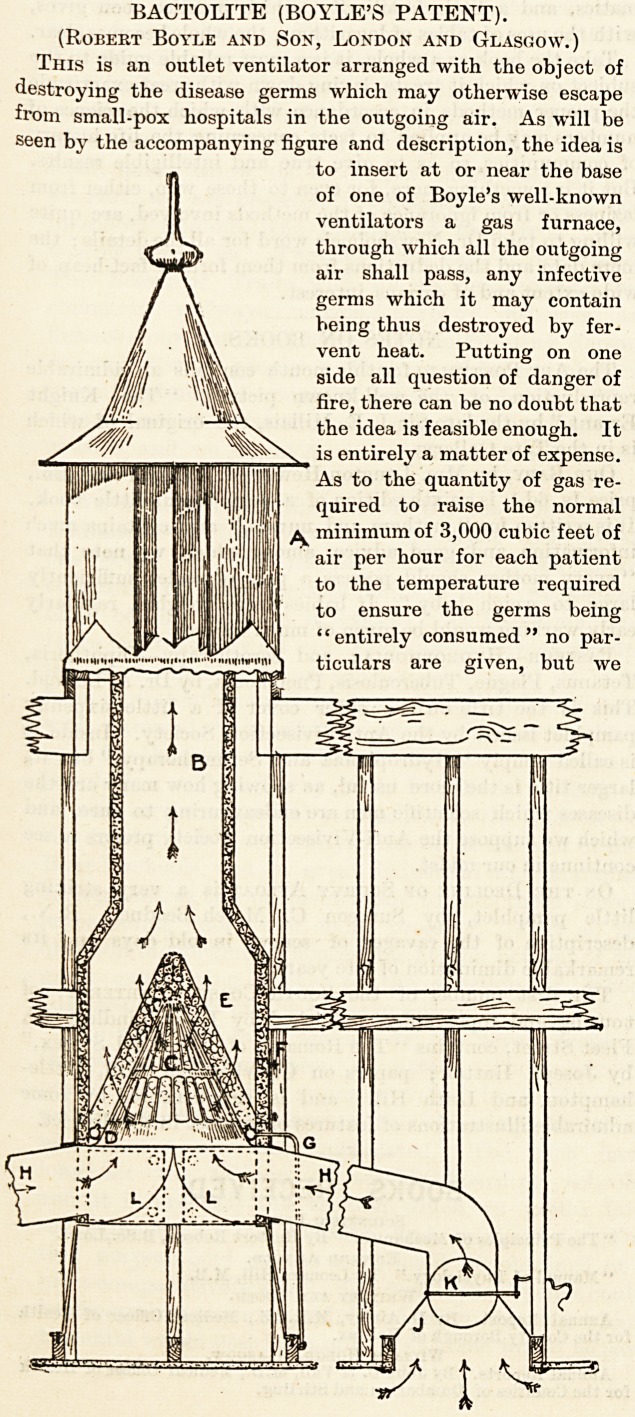# New Appliances and Things Medical

**Published:** 1899-07-22

**Authors:** 


					NEW APPLIANCES AND THINGS MEDICAL.
[We shall be glad to receive, at our Office 28 & 29 Southampton Street, Strand, London, W.O., from the manufacturers, specimens of all new
preparations and appliances which may be brought out from time to time.]
BACTOLITE (BOYLE'S PATENT).
(Robert Boyle and Son, London and Glasgow.)
This is an outlet ventilator arranged with the object of
destroying the disease germs which may otherwise escape
from small-pox hospitals in the outgoing air. As will be
seen by the accompanying figure and description, the idea is
to insert at or near the base
of one of Boyle's well-known
ventilators a gas furnace,
through which all the outgoing
air shall pass, any infective
germs which it may contain
being thus destroyed by fer-
vent heat. Putting on one
side all question of danger of
fire, there can be no doubt that
the idea is feasible enough. It
is entirely a matter of expense.
As to the quantity of gas re-
quired to raise the normal
minimum of 3,000 cubic feet of
air per hour for each patient
to the temperature required
to ensure the germs being
"entirely consumed" no par-
ticulars are given, but we
fancy it must be considerable. Still, if the official views
at present current in regard to the aerial diffusion of
small-pox are correct, that must be looked upon as a
detail. The accompanying diagram and the following
description fully explain the invention :?(a) Boyle's
patent " air-pump" ventilator, made fireproof, (b) Main
extraction shaft, encased in larger shaft, with space
between packed with non-conducting material, (c) Double
grill, with space between filled with perforated asbestos balls,
through which the disease germs pass and are consumed.
Note: More than one of these double grills may, where
required, be fitted one above the other, (d) Ring of
atmospheric burners, the flames from which render the
asbestos balls incandescent, (e) Fireproof chamber contain-
ing grills, (f) Door giving access to chamber for lighting
and other purposes, (g) Gas pipe, (nil) Branch extraction
shafts connected with openings in ceiling. ( j) Cone covering
opening in ceiling, (k) Weighted regulating valve, (L l)
Door giving access to extraction shafts for cleansing purposes.
POCKET SPITTING POT.
(Maw, Sox, and Thompson, 7-12, Aldersgate Street, E.C.)
This new invention should be a great convenience to
phthisical patients, and a prophylactic means of considerable
importance to the public. It consists of a small glass bottle
infundibular in shape, with a large opening at one end,
into which the sputum can be ejected. It passes through
a funnel into the body of the bottle, whence it cannot return
unless the bottle is full up to the level of the bottom of the
funnel. It can be cleared by means of another opening at
the bottom. The fittings are well finished, and there is no
chance of leakage. It is stated to be unnecessary to put any
antiseptics into the receiver, but in cleaning out the bottle
?the contents should be treated with carbolic acid, and the
interior washed out with the same. It can be readily carried
in the pocket, and its use is obviously more cleanly and safe
than that of the pocket handkerchief or spittoon.
HYDROCHLORIDE OF HEROIN.
(F. Bayer and Co., Elberfeld, and 19, St. Dunstan's
Hill, E.C.)
As a substitute for morphia, heroin has given excellent
results, especially in cases of bronchitis, pharyngitis, and other
inflammatory affections of the respiratory tract. Heroin is not,
however, in all cases an easy drug to administer. Messrs.
Bayer have, however, now introduced the hydrochloride of
heroin, which is readily soluble in water, yielding a neutral
solution, and well adapted for subcutaneous injection. The
dose to commence with should be about the twelfth of a
grain.
BACTOLITE (BOYLE'S PATENT).
(Robert Boyle and Son, London and Glasgow.)
This is an outlet ventilator arranged with the object of
destroying the disease germs which may otherwise escape
from small-pox hospitals in the outgoing air. As will be
seen by the accompanying figure and description, the idea is
? to insert at or near the base
of one of Boyle's well-known
ventilators a gas furnace,
Sj, through which all the outgoing
"rT air shall pass, any infective
/ V germs which it may contain
/ being thus destroyed by fer-
L\i ' M\ vent heat. Putting on one
IIi t '\\vff\ side all question of danger of
Mji: fire, there can be no doubt that
the idea is feasible enough. It
V is entirely a matter of expense.
As to the quantity of gas re-
quired to raise the normal
minimum of 3,000 cubic feet of
air per hour for each patient
to the temperature required
to ensure the germs being
"entirely consumed" no par-
| ticulars are given, but we

				

## Figures and Tables

**Figure f1:**